# The Genetic Diversity in *Thereuonema tuberculata* (Wood, 1862) (Scutigeromorpha: Scutigeridae) and the Phylogenetic Relationship of Scutigeromorpha Using the Mitochondrial Genome

**DOI:** 10.3390/insects13070620

**Published:** 2022-07-11

**Authors:** Yong-Mei Yang, Li-Hua Zhang, Yi-Jie Lin, Yi-Meng Zheng, Wan-Ting Jin, Kenneth B. Storey, Dan-Na Yu, Jia-Yong Zhang

**Affiliations:** 1 College of Chemistry and Life Science, Zhejiang Normal University, Jinhua 321004, China; yym0211@zjnu.edu.cn (Y.-M.Y.); 945496914@zjnu.edu.cn (Y.-J.L.); zymeng@zjnu.edu.cn (Y.-M.Z.); jwtjasmine@zjnu.edu.cn (W.-T.J.); 2 Taishun County Forestry Bureau, Wenzhou 325599, China; zlh8701@126.com; 3 Department of Biology, Carleton University, Ottawa, ON K1S 5B6, Canada; kenstorey@cunet.carleton.ca; 4 Key Lab of Wildlife Biotechnology, Conservation and Utilization of Zhejiang Province, Zhejiang Normal University, Jinhua 321004, China

**Keywords:** Scutigeromorpha, mitogenome, phylogenetic relationship, cryptic species

## Abstract

**Simple Summary:**

The sequencing of mitochondrial genomes promotes the study of cryptic species and the phylogenetic relationship of species. Thus far, only one complete mitochondrial genome of Scutigeromorpha is available in the NCBI. In this study, four specimens of *Thereuonema tuberculata* (Scutigeromorpha: Scutigeridae), collected from four different localities of China, were identified, and the four mitochondrial genomes of those were sequenced and annotated. Based on the gene organization and genetic diversity of the mitochondrial genomes, we hypothesized that cryptic species could exist in *T. tuberculata*. We further constructed BI and ML phylogenetic trees to reveal the relationship of Scutigeromorpha.

**Abstract:**

Based on morphological characteristics to make species identification, the cryptic species of the Scutigeromorpha can be greatly underestimated. The mitochondrial genome provides a desirable tool for the biological identifications and the discovery of the cryptic species. The capacity to acquire mitochondrial genome sequences has substantially improved in recent years using next-generation sequencing (NGS) technology. On the basis of the next-generation sequencing, we obtained four complete mitochondrial genomes of *Thereuonema tuberculata* (Wood, 1862) from Nanyang, Henan Province (NY), Nanchang, Jiangxi Province (NC), Jinan, Shandong Province (JN), and Dali, Yunnan Province (DL) in China with GenBank numbers OK513221, OL449685, ON058988 and ON058989, respectively. The lengths of the four mitochondrial genomes ranged from 14,903 to 14,909 bp. The composition and order of genes of the four mitochondrial genomes were identical to the published mitochondrial genome of *Scutigera coleoptrata* (Linnaeus, 1758) (Scutigeromorpha: Scutigerdae). It was the first time that the tandem repeats in the control region were detected in Scutigeromorpha. We also calculated the corrected pairwise genetic distance of four complete mitochondrial genomes of *T. tuberculata*, ranging from 7.7 to 15.2%. The results showed that the *T.* *tuberculata* NC belonged to the typical sample of *T. tuberculata*, and *T. tuberculata* DL was hypothesized as a cryptic species of *T. tuberculata*. Meanwhile, *T. tuberculata* NY and *T. tuberculata* JN were hypothesized as potential cryptic species of *T. tuberculata* in this study. In both BI and ML trees, the monophyly of Scutigeromorpha, Scolopendromorpha, Geophilomorpha, and Lithobiomorpha was forcefully advocated. Moreover, Scutigeromorpha was recovered as the sister clade of (Scolopendromorpha + (Lithobiomorpha + Geophilomorpha)). Four specimens of *T*. *tuberculata* were clustered into one clade, which was the sister to the clade of *S. coleoptrata*.

## 1. Introduction

The class Chilopoda (Latreille, 1817) is one of the four major lineages of myriapods [1]. Six orders of centipedes are currently recognized, including five extant orders of Craterostigmomorpha (Pocock, 1902), Geophilomorpha (Pocock, 1895), Lithobiomorpha (Pocock, 1895), Scolopendromorpha (Pocock, 1895), and Scutigeromorpha (Pocock, 1895), and an extinct order of Devonobiomorpha [2,3]. The debate about the phylogeny of Chilopoda has been a heated discussion in recent years. Based on the morphological features, it was traditionally believed that Chilopoda was divided into two branches, the subclass Notostigmophora (Verhoeff, 1901) (only consisting of the order Scutigeromorpha) and the subclass Pleurostigmophora (Verhoeff, 1901). Meanwhile, the hypothesis of the division of Notostigmophora and Pleurostigmophora had been supported by molecular data [4,5,6,7]. Within Pleurostigmophora, the order Lithobiomorpha is the earliest diverging clade while the order Scolopendromorpha and the order Geophilomorpha cluster into one clade. The position of Craterostigmomorpha is controversial, as they are not strictly anamorphic [1,6,7]. Based on morphological characteristics and molecular evidence, Scutigeromorpha, called house centipede, is an ancient lineage of terrestrial Chilopoda probably diverged in the Silurian [8,9,10], which is a sister group to all remaining centipedes [4,11,12]. There are nearly 95 described species of Scutigeromorpha in the world [8]. These house centipedes are fast predators and prey upon smaller arthropods, and the agility of their movements makes them difficult to capture [13,14]. The main morphological characteristics of house centipedes are the unique position of spiracles on the margins of the tergal plates [5], and compound eyes [14,15]. Scutigeromorpha is divided into three families: Pselliodidae (Kraus, 1955), Scutigerinidae (Attems, 1928) and Scutigeridae (Gervais, 1837), among which the Scutigeridae species are the most widely distributed [1]. The monophyly of the three families is well supported, and Pselliodidae is a sister clade to (Scutigerinidae + Scutigeridae) [8,16]. Thereuoneminae is a subfamily of the family Scutigeridae with two stable clades: one consisting of genera *Allothereua* and *Parascutigera*, and the other consisting of genera *Thereuopoda*, *Thereuonema* and *Thereuopodina* [8,11,17]. *Thereuonema tuberculata* (Wood, 1862) is widespread in temperate and tropical regions around the world [18]. *Thereuonema tuberculata* and *Scutigera coleoptrata* are both predatory or scavenge recently dead arthropods, and they autotomize limbs frequently, as they have exoskeletal rifts along the entire circumference of the trochanter [19,20]. They can be well distinguished, as *S. coleoptrata* has a pair of spine-bristles at the distal end of the first tarsal segment of legs 5 or 6 to 14 but not in *T. tuberculata* [21].

However, there are still many problems in the species identification of Scutigeromorpha. Many species are underestimated with neglecting the extent of ontogenetic and intra- and inter-population variation. As the rationality of the initial classification is poor, it is easy to exhibit polymorphic species with broad geographic distributions and extensive synonymy [17]. Würmli proved that many species of *Brasilophora* and *Pselliodes* described by Bücherl and Chamberlin [22] and a large number of species named by Verhoeff were synonymous [12,23,24,25,26,27]. The number of the Scutigeromorpha species is greatly underestimated, as many Scutigeromorpha species may not yet have been recorded [28]. Based on the traditional morphology, the introduction of molecular data brings great progressive significance to explore the phylogeny, biogeography and taxonomy of Scutigeromorpha [18].

The mitochondrial genomes in Arthropods, a kind of double-stranded circular molecular genome, are generally seen as an informative genetic molecular marker because it includes 22 transfer RNAs (tRNAs), 13 protein-coding genes (PCGs), two ribosomal RNAs (rRNAs), and a relatively large non-coding region with high AT% (also regarded as the A + T rich region or control region) [29,30]. The mitochondrial genome has characteristics as follows: relatively high evolution rates, relatively small genomic size, relatively rare sequence recombination and maternal inheritance [31,32]. Hence, the mitochondrial genome has great potential for serving as a molecular marker of phylogenetic analyses [33,34,35,36,37]. Moreover, the features (gene order, gene copy and size of noncoding regions) and genetic divergence of the mitochondrial genome are used to identify cryptic species [36,38,39,40,41]. As many aspects of Scutigeromorpha’s morphology are highly conserved [12], taxonomic expertise is not sufficient enough to make an accurate species diagnosis. We considered that the cryptic species of the Scutigeromorpha have been greatly underestimated, and the mitochondrial genomes can be feasible for examining closely related species or cryptic species in Scutigeromorpha.

In the present study, we collected four specimens of *T. tuberculata* from four different localities and used the mitochondrial genomes to explore the cryptic species of *T. tuberculata*. For the purpose of discussing the relationship of Scutigeromorpha, relevant phylogenetic analyses and phylogenetic trees (BI and ML tree) about Chilopoda were conducted using four mitochondrial genomes in this study and other Chilopoda mitochondrial genomes already available in GenBank.

## 2. Material and Methods

### 2.1. Sample Collection, Species Identification and DNA Extraction

Four populations of *T. tuberculata* were captured from Nanyang, Henan Province; Nanchang, Jiangxi Province; Jinan, Shandong Province; and Dali, Yunnan Province, China (Table 1). The specimens were examined under an optical stereomicroscope (Nikon SMZ-1500, Japan). We dissected, observed and photographed the head capsule, antenna, epipharynx, hypopharynx, second maxilla, tergal plate, leg, sternites, gonopod, and forcipules of *T. tuberculata.* Based on the external morphologically features, four populations were all morphologically identified as *T. tuberculata* by JY Zhang and deposited in the College of Life Sciences and Chemistry, Zhejiang Normal University, China. Total genomic DNA was extracted from muscle tissue of ambulatorial legs using the Ezup Column Animal Genomic DNA Purification Kit (Sangon Biotech Company, Shanghai, China). Then, the extracted DNA samples were stored at −20 °C until used for PCR and NGS.

### 2.2. COX1 Sequences and Next Generation Sequencing

We used the modified primers LCO1141 (5′-TTTCWACWAAYCAYAAAGAYATYGG-3′) and HCO1849 (5′-TADACTTCWGGRTGDCCRAARAAYCA-3′) [42] to amplify the fragment of the COX1 gene. For details of the procedure and process of polymerase chain reaction (PCR), mainly refer to Zhang et al. [42]. We used normal PCR (product length <3000 bp) in a 50 µL reaction volume. The specific procedure contained 5 µL of 10× *Taq* Buffer (Mg^2+^ plus), 1 µL of MgCl_2_ (25 mM), 4 µL of dNTP (2.5 mM each), 35 µL of ddH_2_O, 2 µL of each primer (10 µM), 0.25 µL of *Taq* polymerase (Takara, Dalian, China) (5 U/µL), and 1 µL of template DNA. The PCR thermal regime comprised 1 cycle of 5 min at 94 °C; 35 cycles of 50 s at 94 °C; 30 s at 51–53 °C; 1 min at 72 °C; and a final cycle of 10 min at 72 °C. COX1 gene was obtained using Sanger sequencing in both directions at Sangon Biotech Company (Shanghai, China). Via using DNASTAR Package v. 6.0 software, all COX1 sequences were manually proofread [43]. Then, we aligned the nucleotide sequences of the partial COX1 by Mega 7.0 to check the similarity [44].

In order to obtain the complete mitochondrial genomes of *T. tuberculata*, the total DNA of each sample, such as the materials for next-generation sequencing (NGS), were dispatched to BGI Tech. Inc. (Shenzhen, China). The KAPA HiFi HotStart PCR Kit (BGI-Shenzhen, China) and protocol were chosen to process the libraries. Based on the shotgun method, samples were sequenced on the Illumina MiSeq Platform to produce 2 × 150 bp paired reads. FASTQC [45,46] was used to perform a quality-adjusted check and trim on raw paired readings. Raw sequence reads for each specimen-specific library were deposited in the BioProject PRJNA842516. We used the clean data to assemble the four mitochondrial genomes of *T. tuberculata* through NOVOPlasty using the default settings [47] and selected *S. coleoptrata* (Scutigeromorpha: AJ507061) and partial COX1 gene of each sample as the reference sequence (seed sequence).

### 2.3. Sequence Analyses and Annotation

We used MITOS (http://mitos.bioinf.uni-leipzig.de/index.py) (accessed on 30 January 2022) to determine the locations of the tRNA genes [48]. Compared with the reference sequence of *S. coleoptrata*, we manually proofread the precise locations of two rRNA genes and thirteen protein-coding genes by using Clustal X [49]. Then, the thirteen PCGs of four mitochondrial genomes were translated into amino acids according to the invertebrate mitochondrial genetic codes by Mega 7.0 [44]. In addition, we used the Mega 7.0 to calculate the corrected genetic distance. All positions containing gaps and missing data were removed. Cloverleaf secondary structures were forecasted and identified using MITOS and ARWEN (http://130.235.244.92/ARWEN/index.html) (accessed on 30 January 2022) [50]. Then, they were drawn on Forna (http://rna.tbi.univie.ac.at/, accessed on 30 January 2022) [51]. The codon skews, nucleotide composition, and relative synonymous codon usage (RSCU) were obtained using PhyloSuite v1.2.2 [52]. We used the formula: AT skew = (A − T)/(A + T), GC skew = (G − C)/(G + C) to calculate the GC skews and AT skews [53]. Four mitochondrial genome maps were drawn online using CGView server V 1.0 (http://cgview.ca/) (accessed on 25 February 2022) [54]. Tandem repeat sequences within the control region were detected using the TRDB (https://tandem.bu.edu/trf/trf.html) (accessed on 30 January 2022) [55].

### 2.4. Phylogenetic Analyses

To date, the only mitochondrial genome of Chilopoda, *Scutigera coleoptrata* [56], was public in the NCBI. In this study, the mitochondrial genomes of 17 chilopods including *T. tuberculata*, *S. coleoptrata* [56], two Geophilomorpha species [57], four Scolopendromorpha species [58,59] as well as three Lithobiomorphas species [60,61,62] were selected as the ingroup taxa in order to investigate the phylogenetic relationships within Chilopoda. Since the diplopod was considered as a more distant and suitable outgroup to root the centipede tree [8,11], *Anaulaciulus koreanus* (Helminthomorpha: Julidae) [63] and *Spirobolus bungii* (Helminthomorpha: Spirobolidae) (MT767838) were chosen as the outgroup taxa for phylogenetic analyses. The information on all mitochondrial genomes in this study were listed in Table 2.

All phylogenetic analyses were performed using the 1st + 2nd + 3rd codons. For the phylogeny, the 13 PCGs were obtained from 19 mitochondrial genomes using PhyloSuite v1.1.16 [52] and used for the DNA alignment of sequences by MAFFT v. 7.475 [64]. According to the default parameters, the relevantly conserved regions were detected by Gblock 0.91b [65]. Finally, the 13 PCGs were combined to a single line using concatenate sequence, which was performed in the PhyloSuite v1.1.16. Based on the Bayesian information criterion (BIC), the program PartionFinder 1.1.1 [66] was employed to identify the optimal partitioning strategies and best substitution models. The partition schemes and best-fit models selected for each data set are provided in Table S1. We used the best-fit models to construct BI and ML analyses. The BI analysis was performed in MrBayes 3.2 [67] for 10 million generations. Moreover, in order to achieve the convergence of the independent runs, the mean standard deviation of split frequencies in MrBayes 3.2 was set to less than 0.01. The first 25% of sampled generations was removed as burn-in. The ML analysis was performed in IQ-TREE [68] software using a maximum likelihood approach. The 1000 ultrafast bootstrap replicates were used in the IQ-TREE software package. The phylogenetic trees were illustrated using FigTree v1.4.3 [69].

## 3. Results and Discussion

### 3.1. Species Identification

The specimens of four populations from four locations showed the morphological characteristics of a yellow body, with a length of 26.3 mm on average, the width of the head narrower than the width of the abdomen, a pair of compound eyes on the sides of the head, a fixed number of 15 pairs of elongated yellow legs in the adults, the length of legs increased from the anterior to the posterior pairs, multi-annulated antennae on the head consisting of a few hundred ring-like articles, a spiracle opened on the posterior part of the tergites, and no raised saddle surrounding the spiracle. Some black pigmentations surrounded the spiracle [20]. There was no spine on the distal end of a first tarsal segment of legs 6–14 [21]. Based on the COX1 and their morphological characteristics [20], the species from the four localities were all identified as *Thereuonema tuberculata*.

### 3.2. Mitochondrial Genome Organization and Composition

#### 3.2.1. General Features of Mitochondrial Genomes

**Figure 1:**
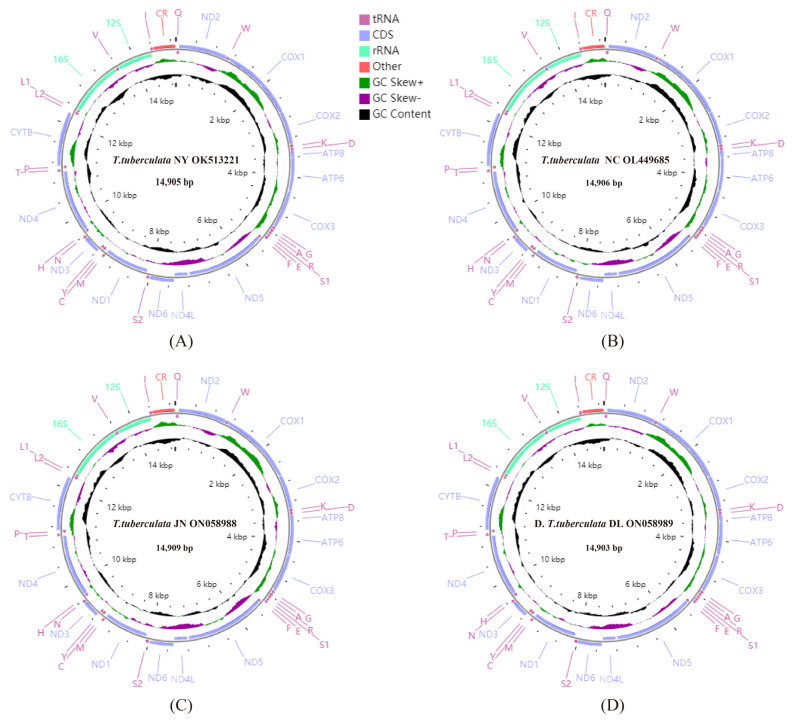
Mitochondrial genome maps of *T. tuberculata* NY (**A**), *T. tuberculata* NC (**B**), *T. tuberculata* JN (**C**), and *T. tuberculata* DL (**D**). The first circle shows the gene map (PCGs, rRNAs, tRNAs, and the AT-rich region). The genes shown outside the map are coded on the majority strand (J strand), whereas the genes inside the map are coded on the minority strand (N strand). The second circle shows the GC skew, and the third shows the GC content. GC content and GC skew are plotted as the deviation from the average value of the entire sequence.

The lengths of whole mitochondrial genomes of *T. tuberculata* NY, *T. tuberculata* NC, *T. tuberculata* JN, *T. tuberculata* DL were 14,905, 14,906, 14,909, and 14,903 bp, respectively (Figure 1). The four mitochondrial genomes had the same gene order and gene composition, which were consistent with the other sequenced Scutigeromorpha genome (*S. coleoptrata*) [29].

Some intergenic regions and overlaps were detected in the four mitochondrial genomes. The mitochondrial genomes of *T. tuberculata* NY, *T. tuberculata* NC, *T. tuberculata* JN, and *T. tuberculata* DL contained eight, nine, nine, and seven intergenic regions with lengths of 69, 68, 68 and 62 bp in total, respectively. Four mitochondrial genomes contained 12, 12, 11, and 12 overlaps with lengths of 37, 43, 42 and 42 bp in total, respectively. The overlaps of four mitochondrial genomes ranged from 1 to 15 bp, and the intergenic regions of four mitochondrial genomes ranged from 1 to 35 bp. Moreover, between ND5 and ND4L, the longest intergenic region of four mitochondrial genomes was found, with lengths of 35, 33, 33, and 29 bp, respectively.

The length, A + T content, AT skew and GC skew of corresponding regions of each specimen from different localities were calculated and are shown in Table 3. There were strong A + T biases in the mitochondrial genomes of the *T. tuberculata* NY, *T. tuberculata* NC, *T. tuberculata* JN, and *T. tuberculata* DL, with values of 71.8, 71.9, 71.7, and 71.0%, respectively (Table 3), which were higher than the percentage found in *S. coleoptrata* (69.41%) [29]. All four mitochondrial genomes presented a positive AT skew and a negative GC skew.

#### 3.2.2. Protein-Coding Genes and Codon Usages

Nine PCGs (ND2, COX1, COX2, ATP8, ATP6, COX3, ND6, ND3, and Cyt b) of four mitochondrial genomes were encoded on the J strand, whereas the residual four PCGs (ND5, ND4L, ND1, ND4) were encoded on the N strand (Table S2). The total sizes of the 13 PCGs of *T. tuberculata* NY, *T. tuberculata* NC, *T. tuberculata* JN, and *T. tuberculata* DL were 11,079, 11,082, 11,082, and 11,088 bp, respectively (Table 3). For the thirteen PCGs, the A + T content was 71.1, 70.9, 70.8 and 70.0% in *T. tuberculata* NY, *T. tuberculata* NC, *T. tuberculata* JN, and *T. tuberculata* DL, respectively. The nucleotide skews were positive for AT and negative for GC in four mitochondrial genomes. The shortest PCG was the ATP8 (156 bp) in the four mitochondrial genomes, whereas the longest PCG was the ND5 with 1713 bp in *T. tuberculata* NY, 1717 bp in *T. tuberculata* NC, *T. tuberculata* JN, and 1716 bp in *T. tuberculata* DL, respectively.

Among the total mitochondrial genomes of *T. tuberculata* from four locations, there were 12 mitochondrial PCGs that used the typical invertebrate initiation codon ATN (N represents A, G, C, or T), whereas COX1 used TTG and ND5 used TTA as the initiation codon in specimens from four localities, ND6 used TTA as the initiation codon in *T. tuberculata* NY, *T. tuberculata* NC, *T. tuberculata* JN. In the majority of PCGs, the conventional terminal codons TAA or TAG were detected. However, the truncated stop codons TA were found in COX2 (specimens from four localities), COX3 (specimens from four localities), ND1 (*T. tuberculata* DL) and incomplete terminal codons T were found in ND3 (specimens from four localities), ND4 (specimens from four localities), ND5 (*T. tuberculata* NC, *T. tuberculata* JN), and ND6 (*T. tuberculata* NY, *T. tuberculata* NC, *T. tuberculata* JN). In metazoan mitochondrial genomes, the incomplete stop codons are proposed to be generated by post-transcriptional polyadenylation during the mRNA maturation process [70,71]. Meanwhile, T is present more often than TA [72].

**Figure 2:**
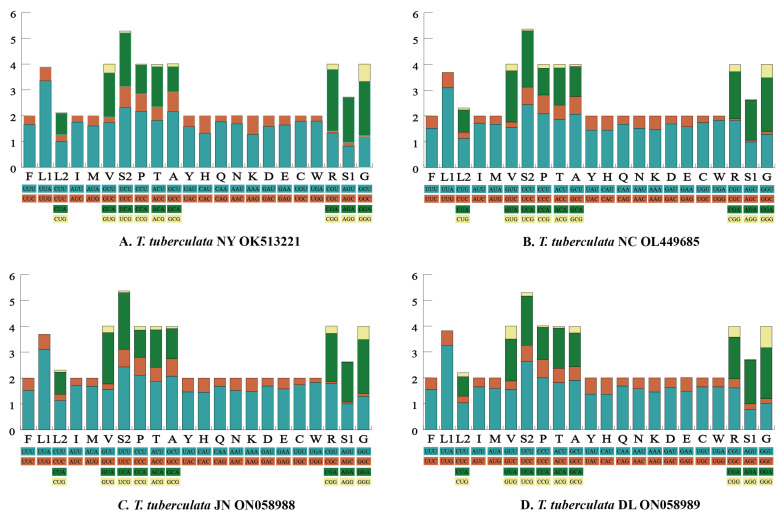
The relative synonymous codon usage (RSCU) of the 13 protein-coding genes. Codon families are provided on the *x*-axis along with the different combinations of synonymous codons that code for that amino acid. RSCU is defined on the *y*-axis.

The RSCU of each mitochondrial genome is shown in Figure 2. We found that the main codons used in four mitochondrial genomes were highly similar. The most commonly used codons (≥245) in the PCGs of the four mitochondrial genomes were AUU (Ile), UUA (Leu), and UUU (Phe). Meanwhile, the usage of AUU (Ile) was high, with a frequency of ≥302. In the PCGs of *S. coleoptrata* [56], the usages of AUU (Ile), UUA (Leu) and UUU (Phe) were slightly lower, with a frequency of 262, 233 and 225 times, respectively. By contrast, codons comprising a third codon G or C in the PCGs of four mitochondrial genomes were used rarely, such as CCG (Pro), CGC (Arg), and AGG (Ser1). In the PCGs of *S. coleoptrata*, the usage of the CGC (Arg) was rare [56], with a frequency of 0 times.

#### 3.2.3. Ribosomal RNAs, Transfer RNAs and Hairpin Structures

Two rRNAs were both on the minor strand (Table S2). As in the other sequenced Scutigeromorpha mitochondrial genome (*S. coleoptrata*), the 16S rRNA gene was positioned between trnL1 (CUA) and trnV, with a length of 1186, 1188, 1193, and 1206 bp in *T. tuberculata* NY, *T. tuberculata* NC, *T. tuberculata* JN, *T. tuberculata* DL, respectively. The size of the 12S rRNA gene separated the trnV and trnI had been estimated to be 764, 766, 763, and 765 bp in *T. tuberculata* NY, *T. tuberculata* NC, *T. tuberculata* JN, *T. tuberculata* DL, respectively. We recovered that the AT skew values of the two rRNAs in four mitochondrial genomes were −0.028, −0.039, −0.030 and −0.022, respectively, whereas the AT skew of two rRNAs in the mitochondrial genome of *S. coleoptrata* [56] were −0.055. Meanwhile, the GC skew values were highly positive, with the values of 0.368, 0.392, 0.375 and 0.343, respectively (Table 3), and the GC skew value of two rRNAs in *S. coleoptrata* [56] was 0.365.

Additionally, all of the trefoil structures of tRNA genes are displayed in Figure S1. However, not all of the second structure of the tRNA genes was intact. For instance, we found the trnS1 of four mitochondrial genomes lacked the DHU arm, which is a common condition in metazoan mitochondrial genomes [29,62]. We also found that the trnE, trnF and trnC of four mitochondrial genomes lacked the TΨC loops, as well as trnN of *T. tuberculata* NC and *T. tuberculata* DL. Meanwhile, the trnT of *T. tuberculata* JN had lost the T arm. Compared to the normal structures, these lacks have lessened the translational activity [73]. Some mismatches were present in the hairpin structures in the T arms and the AA arm. For the T arm, U-U in trnW of the four mitochondrial genomes and A-A in trnR (*T. tuberculata* NC and *T. tuberculata* JN) were mismatched. For the AA arm, U-U in *trnD* (*T. tuberculata* NY and *T. tuberculata* DL) and trnE (*T. tuberculata* NY, *T. tuberculata* NC and *T. tuberculata* DL), U-C in trnN (*T. tuberculata* DL) were mismatched. These mismatched pairs may affect aminoacylation and translation [74].

#### 3.2.4. A + T Rich Region

The putative non-coding region was positioned between the trnI and trnQ genes in four mitochondrial genomes. The length of the A + T-rich region of *T. tuberculata* NY, *T. tuberculata* NC, *T. tuberculata* JN, and *T. tuberculata* DL was 461, 463, 463 and 439 bp, respectively, which was lower than the mitochondrial genome of *S. coleoptrata* (479 bp) [56]. In the mitochondrial genomes of *T. tuberculata* NY, *T. tuberculata* NC, *T. tuberculata* JN, and *T. tuberculata* DL, the A + T content in the control regions was much higher than other regions in the mitochondrial genomes, with a value of 80.3, 83.4, 82.5, and 81.5%, respectively.

**Figure 3:**
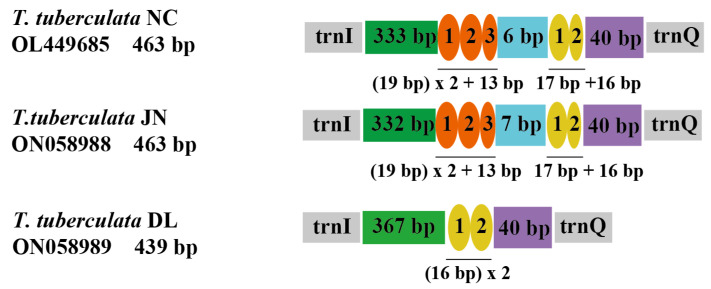
Organizations of the repeat regions in the A + T rich region of the *T. tuberculata* NC, *T. tuberculata* JN, and *T. tuberculata* DL. Ovals with different colors indicate tandem repeat sequences. The colored box shows the non-repeat region.

The organization of repeat regions in the A + T-rich region of the mitochondrial genomes are shown in Figure 3. Repeat regions were observed in *T. tuberculata* NC, *T. tuberculata* JN, and *T. tuberculata* DL, whereas a non-repeat region was found in *T. tuberculata* NY. The A + T-rich region of *T. tuberculata* NC possessed two interspersed repeat regions. One with 19 bp was repeated 2.6 times, and the other with 17 bp was repeated 1.9 times, representing a good example of the TDRL model (Table S3). The former repeat region had lost a partial fragment in the third after three repeats of the 19 bp copy, whereas the latter repeat region had lost a partial fragment in the second after two repeats of the 17 bp copy. Meanwhile, the A + T-rich region of *T. tuberculata* JN showed similar organization to the *T. tuberculata* NC. However, in the A + T-rich region of *T. tuberculata* DL, only one repeat region with two similar copies of a 16 bp tandem repeat was identified. There were strong A + T biases of 100% in the repeat region of *T. tuberculata* NC, *T. tuberculata* JN and *T. tuberculata* DL. A positive AT skew and a negative AT skew were detected in two repeat regions of the A + T-rich region of *T. tuberculata* NC and *T. tuberculata* JN, respectively. Meanwhile, the repeat region of the mitochondrial A + T region of the *T. tuberculata* DL showed a positive AT skew.

An interesting feature in the mitochondrial genome of *Scolopendra mutilans* L. Koch, 1878 (Scolopendromorpha, Scolopendridae), nine simple sequence repeats (SSRs), including three mono-nucleotides, five dinucleotides, and one trinucleotide, as well as two compound SSRs, was revealed. Repeat regions of the mitochondrial control region also existed in other Chilopoda species. Five interspersed repeat regions were identified in the putative control region of the *Mecistocephalus marmoratus* (Geophilomorpha: Mecistocephalidae) (KX774322). Moreover, four interspersed repeat regions were identified in the putative control region of the *Lithobius forficatus* (Lithobiomorpha: Lithobiidae) [62]. The control region in mitochondrial genomes may represent starting points for the mtDNA duplication process and regulates transcription plus replication [30,75].

### 3.3. The Corrected Pairwise Genetic Distance of T. tuberculata

The corrected pairwise genetic distance of four complete mitochondrial genomes and partial COX1 of *T. tuberculata* is listed in the Table 4. The genetic distance between four populations from four different localities ranged from 7.7 to 15.2%, with an average of 12.1% (Table 4). The genetic distance between the *T. tuberculata* NC and *T. tuberculata* NY was 9.70%, between the *T. tuberculata* NC and *T. tuberculata* JN was 7.7%, whereas the genetic distance between the *T. tuberculata* NC and *T. tuberculata* DL was 15.0%. Meanwhile, the genetic distance between the *T. tuberculata* DL and *T. tuberculata* NY was 15.2%, whereas the genetic distance between the *T. tuberculata* DL and *T. tuberculata* JN was 15.1%. In addition, the calculated genetic distance between *T. tuberculata* NY and *T. tuberculata* JN was 10.0%.

The genetic distance between species of centipedes was useful for species delimitation of Scutigeromorpha. Wesener discovered that the genetic distance of species of *Cryptops* in different geographic localities was 13.7–22.2%, which enabled the detection of potential cryptic lineages in the widespread German species [76]. In the research of Siriwut et al., the results showed a corrected mean distance for COX1 between 13.8–21.3% among interspecific populations [77]. A clear gap was also found in the genetic distance for COX1 between and within species, as the average genetic distance within species was 6.4 and the highest was 9.1%, whereas the average genetic distance was 14.8% and the lowest was 13.5% between species [78]. Hence, our results hypothesized that *T. tuberculata* DL was a cryptic species of the *T. tuberculata.* Meanwhile, *T. tuberculata* NY and *T. tuberculata* JN were hypothesized as potential cryptic species of the *T. tuberculata* in this study. However, a finer geographical sampling of all taxa would be necessary to further explore the cryptic species of *T. tuberculata*.

### 3.4. Phylogenetic Analyses

**Figure 4:**
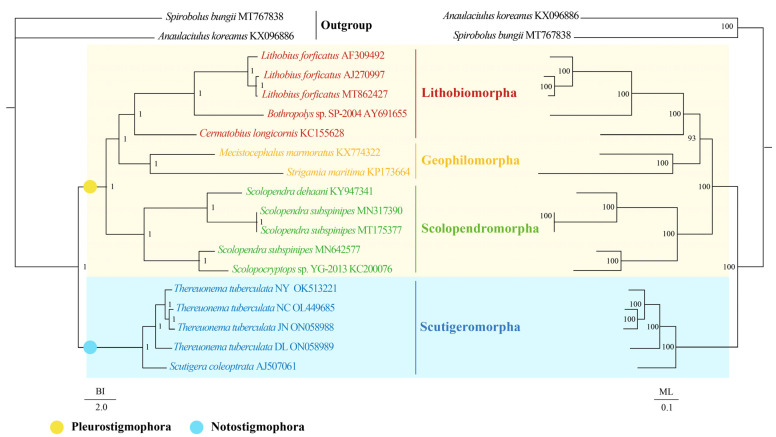
Phylogenetic relationships of Scutigeromorpha inferred from BI analysis (**left**) and ML analysis (**right**) based on 13 mitochondrial protein-coding genes including 17 Chilopoda species. Two species of Diplopoda (*A. koreanus* and *S. bungii*) were chosen as outgroups. The GenBank accession numbers of all species are shown in the figure. The numbers above the branches specify posterior probabilities as determined from BI (**left**) and bootstrap percentages from ML (**right**).

The topologies of the BI and ML phylogenetic analyses were congruent except for the outgroups (Figure 4). The results presented that both BI and ML trees within Chilopoda divided into two branches: (1) the clade of Scutigeromorpha was the most basal clade, and (2) the other orders united into another branch. The results support the hypothesis of Notostigmophora and the Pleurostigmophora [4,5,6,7,79,80]. Moreover, the monophyly of the ordinal groups Scutigeromorpha, Scolopendromorpha, Geophilomorpha, and Lithobiomorpha was forcefully advocated in both BI and ML trees. Then, the Scolopendromorpha was supported as a sister clade of (Lithobiomorpha + Geophilomorpha), consistent with the research of Hu et al. [59], although it was in conflict with a well-corroborated scheme of interrelationships for Pleurostigmophora that Lithobiomorpha was the sister clade of (Scolopendromorpha + Geophilomorpha) [4,5,6,7,79,80]. For Scutigeromorpha, we found that all *T. tuberculata* clustered together and the clade of (((*T. tuberculata* NC + *T. tuberculata* JN) + *T. tuberculata* NY) + *T. tuberculata* DL) was the sister clade of *S. coleoptrata.* With Chilopoda being poorly represented and few known data on the mtDNA of the Scutigeromorpha and Craterostigmomorph. The intra-ordinal and inter-ordinal relationships among Scutigeromorpha are still challenging to determine. Further morphological and molecular data are required to demonstrate a more exact phylogenetic relationship among Scutigeromorpha.

## 4. Conclusions

In this scientific study, we successfully obtained the four complete mitochondrial genomes of *Thereuonema tuberculata* from four different localities to discuss the cryptic species that existed in *T. tuberculata* and the phylogenetic relationship of Scutigeromorpha. Four complete mitochondrial genomes showed the difference in genome composition and genetic distance. Meanwhile, several control region tandem repeats were found in Scutigeromorpha. According to the genetic distance and genome composition, we hypothesized that (1) *T. tuberculata* DL could be recognized as a cryptic species of *T. tuberculata*; (2) *T. tuberculata* NY and *T. tuberculata* JN were hypothesized as potential cryptic species of *T. tuberculata*. In the phylogenetic analyses, the monophyly of the four main Chilopoda orders (Scutigeromorpha, Scolopendromorpha, Geophilomorpha, and Lithobiomorpha) was recovered. Scolopendromorpha was the sister clade of (Lithobiomorpha + Geophilomorpha), and Scutigeromorpha was the basal clade of Chilopoda. However, the sampling was limited, and formal molecular species delimitation analysis has not yet been conducted. Thus, further analyses would be used to explore the cryptic species of *T. tuberculata.*

## Figures and Tables

**Table 1:** Information on specimen sources of the samples used in this study and NCBI GenBank accession numbers.

Species	Specimen No.	Sampling Localities	Accession Number
*Thereuonema tuberculata*	HNYY01	Nanyang, Henan, China	OK513221
	JXYY02	Nanchang, Jiangxi, China	OL449685
	SDYY03	Jinan, Shandong, China	ON058988
	YNYY11	Dali, Yunnan, China	ON058989

**Table 2:** Species used to construct the phylogenetic relationships along with GenBank accession numbers.

Class	Order	Family	Genus	Species	Length (bp)	GenBank No.	References
Diplopoda	Helminthomorpha	Julidae	*Anaulaciulus*	*Anaulaciulus koreanus*	14,916	KX096886	[63]
		Spirobolidae	*Spirobolus*	*Spirobolus bungii*	14,879	MT767838	Direct Submission
Chilopoda	Scolopendromorpha	Cryptopidae	*Scolopocryptops*	*Scolopocryptops* sp. 1 YG-2013	15,119	KC200076	[58]
		Scolopendridae	*Scolopendra*	*Scolopendra mutilans*	15,011	MN317390	[59]
				*Scolopendra mutilans*	15,030	MT175377	Unpublished
				*Scolopendra subspinipes*	14,637	MN642577	Unpublished
				*Scolopendra dehaani*	14,538	KY947341	Unpublished
	Lithobiomorpha	Henicopidae	*Cermatobius*	*Cermatobius longicornis*	16,833	KC155628	[61]
		Ethopolyidae	*Bothropolys*	*Bothropolys* sp. SP-2004	15,139	AY691655	Direct Submission
		Lithobiidae	*Lithobius*	*Lithobius forficatus*	15,437	AJ270997	[60]
				*Lithobius forficatus*	15,695	AF309492	[62]
				*Lithobius forficatus*	15,038	MT862427	Unpublished
	Geophilomorpha	Mecistocephalidae	*Mecistocephalus*	*Mecistocephalus marmoratus*	15,279	KX774322	Unpublished
		Linotaeniidae	*Strigamia*	*Strigamia maritima*	14,983	KP173664	[57]
	Scutigeromorpha	Scutigeridae	*Scutigera*	*Scutigera coleoptrata*	14,922	AJ507061	[56]
			*Thereuonema*	*Thereuonema tuberculata* NY	14,905	OK513221	This study
				*Thereuonema tuberculata* NC	14,906	OL449685	This study
				*Thereuonema tuberculata* JN	14,909	ON058988	This study
				*Thereuonema tuberculata* DL	14,903	ON058989	This study

**Table 3:** Base composition of the mitochondrial genomes of the *T. tuberculata* from four localities.

Region	*T. tuberculata* NY				*T. tuberculata* NC				*T. tuberculata* JN				*T. tuberculata* DL			
	Length (bp)	A + T(%)	AT Skew	GC Skew	Length (bp)	A + T(%)	AT Skew	GC Skew	Length (bp)	A + T(%)	AT Skew	GC Skew	Length (bp)	A + T(%)	AT Skew	GC Skew
Mito	14,905	71.8	0.005	−0.287	14,906	71.9	0.013	−0.292	14909	71.7	0.018	−0.303	14,903	71.0	0.01	−0.273
PCGs	11,079	71.1	0.001	−0.287	11,082	70.9	0.010	−0.288	11082	70.8	0.017	−0.305	11,088	70.0	0.009	−0.273
rRNAs	1950	73.2	−0.028	0.368	1954	73.1	−0.039	0.392	1956	72.8	−0.03	0.375	1971	73.1	−0.022	0.343
tRNAs	1376	73.3	0.010	−0.196	1374	73.7	0.019	−0.190	1374	73.0	0.034	−0.206	1377	72.7	0.026	−0.184
A + T-rich region	461	80.3	0.022	−0.143	463	83.4	0.005	−0.143	463	82.5	0.005	−0.086	439	81.5	0.006	−0.136

**Table 4:** Corrected pairwise distance of the complete mitochondrial genomes (left) and partial COX1 genes (right) of *T. tuberculata* from four localities.

Sample	Complete Mitochondrial Genomes/Partial COX1 Genes			
	*T. tuberculata* NY	*T. tuberculata* NC	*T. tuberculata* JN	*T. tuberculata* DL
*T. tuberculata* NY				
*T. tuberculata* NC	0.097/0.103			
*T. tuberculata* JN	0.100/0.116	0.077/0.073		
*T. tuberculata* DL	0.152/0.145	0.150/0.140	0.151/0.135	

## Data Availability

The data supporting the findings of this study are openly available in the National Center for Biotechnology Information (https://www.ncbi.nlm.nih.gov) (accessed on 21 March 2022), accession numbers: OK513221, OL449685, ON058988 and ON058989. Raw sequence reads for each specimen-specific library were deposited in the BioProject PRJNA842516.

## Supplementary Materials

The following are available online at https://www.mdpi.com/article/10.3390/insects13070620/s1, Figure S1: Inferred secondary structures of the tRNA genes in *T. tuberculata* NY (A), *T. tuberculata* NC (B), *T. tuberculata* JN (C), and *T. tuberculata* DL (D). Table S1: The partition schemes and best-fitting models were selected. Table S2: Location of features in the mitochondrial genomes of the *T. tuberculata* from four localities. Table S3: Statistics of tandem repeats in the control regions of *T.* *tuberculata* NC, *T.* *tuberculata* JN, and *T.* *tuberculata* DL.
