# Supplementary material for: The Genetic Diversity in Thereuonema tuberculata (Wood, 1862) (Scutigeromorpha: Scutigeridae) and the Phylogenetic Relationship of Scutigeromorpha Using the Mitochondrial Genome
Source: Insects. 2022 Jul 11;13(7):620. doi: 10.3390/insects13070620 (PMC9320382; doi:10.3390/insects13070620)
Supplement: Supplementary file 1 [file insects-13-00620-s001.zip › Table S1 Partition schemes and best-fitting models.pdf]

Table S1. The partition schemes and best-fitting models selected.

Nucleotide Sequence Alignments		
**Subset**	**Subset Partitions**	**Best Model**
Partition 1	ATP8_pos1, ATP6_pos1, ND3_pos1, ND2_pos1, ND6_pos1	GTR+I+G
Partition 2	COX2_pos2, COX3_pos2, Cyt b_pos2, ATP6_pos2	TVM+G
Partition 3	COX1_pos3, COX2_pos3, Cyt b_pos3, COX3_pos3, ATP6_pos3	HKY+I+G
Partition 4	ATP8_pos2, ND3_pos2, ND6_pos2, ND2_pos2	TVM+I+G
Partition 5	ATP8_pos3, ND3_pos3, ND2_pos3, ND6_pos3	TRN+G
Partition 6	COX1_pos1, COX2_pos1, Cyt b_pos1, COX3_pos1	GTR+I+G
Partition 7	COX1_pos2	TVM+I+G
Partition 8	ND4L_pos1, ND1_pos1, ND5_pos1, ND4_pos1	GTR+I+G
Partition 9	ND4L_pos2, ND1_pos2, ND5_pos2, ND4_pos2	GTR+I+G
Partition 10	ND1_pos3, ND5_pos3, ND4_pos3, ND4L_pos3	HKY+G
