# Supplementary material for: The Genetic Diversity in Thereuonema tuberculata (Wood, 1862) (Scutigeromorpha: Scutigeridae) and the Phylogenetic Relationship of Scutigeromorpha Using the Mitochondrial Genome
Source: Insects. 2022 Jul 11;13(7):620. doi: 10.3390/insects13070620 (PMC9320382; doi:10.3390/insects13070620)
Supplement: Supplementary file 1 [file insects-13-00620-s001.zip › Table S2. Location of features in the mtDNA.pdf]

Table S2. Location of features in the mitochondrial genomes of the *T. tuberculata* from four localities.

Gene	Strand	Anticodon	*T.tuberculata* NY		*T.tuberculata* NC		*T.tuberculata* JN		*T.tuberculata* DL	
			Position	Start/stop codon	Position	Start/stop codon	Position	Start/stop codon	Position	Start/stop codon
trnQ	N	UUG	1-68		1-68		1-68		1-68	
ND2	J		70-1065	ATT/TAG	70-1065	ATT/TAG	70-1065	ATT/TAG	70-1065	ATT/TAG
trnW	J	UCA	1064-1126		1064-1126		1064-1126		1064-1126	
COX1	J		1126-2661	TTG/TAA	1126-2661	TTG/TAA	1126-2661	TTG/TAA	1126-2661	TTG/TAA
COX2	J		2665-3344	ATG/TA	2665-3344	ATG/TA	2665-3344	ATG/TA	2665-3344	ATG/TA
trnK	J	CUU	3345-3413		3345-3413		3345-3413		3345-3413	
trnD	J	GUC	3414-3475		3414-3475		3414-3475		3414-3476	
ATP8	J		3476-3631	ATT/TAG	3476-3631	ATT/TAG	3476-3631	ATT/TAG	3477-3632	ATT/TAG
ATP6	J		3625-4299	ATG/TAA	3625-4299	ATG/TAA	3625-4299	ATG/TAA	3626-4300	ATG/TAA
COX3	J		4299-5086	ATG/TA	4299-5086	ATG/TA	4299-5086	ATG/TA	4300-5087	ATG/TA
trnG	J	UCC	5086-5146		5086-5146		5086-5146		5087-5147	
trnA	J	UGC	5147-5209		5147-5209		5147-5209		5148-5210	
trnR	J	UCG	5210-5271		5210-5271		5210-5271		5211-5272	
trnS1 (AGC)	J	GCU	5272-5328		5272-5329		5272-5328		5273-5328	
trnE	J	UUC	5328-5387		5328-5387		5327-5386		5327-5388	
trnF	N	GAA	5387-5446		5387-5446		5387-5446		5389-5448	
ND5	N		5446-7158	TTA/TAA	5441-7157	TTA/T	5441-7157	TTA/T	5448-7163	TTA/TAG
ND4L	N		7194-7478	ATG/TAA	7191-7475	ATG/TAA	7191-7475	ATG/TAA	7193-7477	ATG/TAA
ND6	J		7496-7994	TTA/T	7493-7991	TTA/T	7493-7991	TTA/T	7499-8002	ATT/TAA
trnS2 (UCA)	J	UGA	8004-8072		8001-8069		8001-8069		8001-8069	
ND1	N		8063-9016	ATC/TAA	8060-9013	ATC/TAA	8060-9013	ATC/TAA	8064-9019	ATA/TA
trnM	J	CAU	9009-9070		9006-9067		9006-9067		9005-9067	
trnC	N	GCA	9070-9129		9069-9128		9069-9128		9067-9126	
trnY	N	GUA	9130-9190		9129-9189		9129-9189		9127-9188	
ND3	J		9192-9543	ATT/T	9191-9542	ATT/T	9191-9542	ATT/T	9190-9541	ATC/T
trnN	J	GUU	9544-9607		9543-9605		9543-9606		9542-9604	
trnH	N	GUG	9608-9671		9605-9667		9606-9668		9605-9668	
ND4	N		9672-11007	ATG/T	9668-11003	ATG/T	9669-11004	ATG/T	9669-11004	ATG/T
trnT	J	UGU	11010-11070		11006-11066		11007-11067		11007-11066	
trnP	N	UGG	11068-11129		11064-11124		11065-11125		11064-11124	
Cyt b	J		11131-12246	ATG/TAA	11126-12241	ATG/TAA	11127-12242	ATG/TAA	11130-12245	ATG/TAA
trnL2 (UUA)	N	UAA	12247-12308		12242-12303		12243-12304		12246-12307	
trnL1 (CUA)	N	UAG	12309-12361		12304-12357		12305-12357		12308-12361	
16S rRNA	N		12362-13547		12358-13545		12358-13550		12362-13567	
trnV	N	UAC	13548-13616		13546-13614		13551-13619		13568-13636	
12S rRNA	N		13617-14380		13615-14380		13620-14382		13637-14401	
trnI	J	GAU	14381-14444		14381-14443		14383-14446		14402-14464	
CR			14445-14905		14444-14906		14447-14909		14465-14903	
