# Supplementary material for: The Genetic Diversity in Thereuonema tuberculata (Wood, 1862) (Scutigeromorpha: Scutigeridae) and the Phylogenetic Relationship of Scutigeromorpha Using the Mitochondrial Genome
Source: Insects. 2022 Jul 11;13(7):620. doi: 10.3390/insects13070620 (PMC9320382; doi:10.3390/insects13070620)
Supplement: Supplementary file 1 [file insects-13-00620-s001.zip › Table S3. Statistics of TDRs in the CRS .pdf]

Table S3. Statistics of tandem repeats in the control regions of *T. tuberculata* NC, *T. tuberculata* JN, *T. tuberculata* DL.

Species	Size(bp)	TDRs	Positions	Consensus size (bp)	Copy number	Percent Matches (%)	AT%	AT-Skew
T. tuberculata NC	463	1	334-384	19	2.6	75	100	-0.172
		2	391-423	17	1.9	93	100	0.393
T. tuberculata JN	463	1	333-383	19	2.6	75	100	-0.172
		2	391-423	17	1.9	93	100	0.393
T. tuberculata DL	439	1	368-399	16	2.0	93	100	0.313
