# Supplementary figures and images for: The Genetic Diversity in Thereuonema tuberculata (Wood, 1862) (Scutigeromorpha: Scutigeridae) and the Phylogenetic Relationship of Scutigeromorpha Using the Mitochondrial Genome
Source: Insects. 2022 Jul 11;13(7):620. doi: 10.3390/insects13070620 (PMC9320382; doi:10.3390/insects13070620)

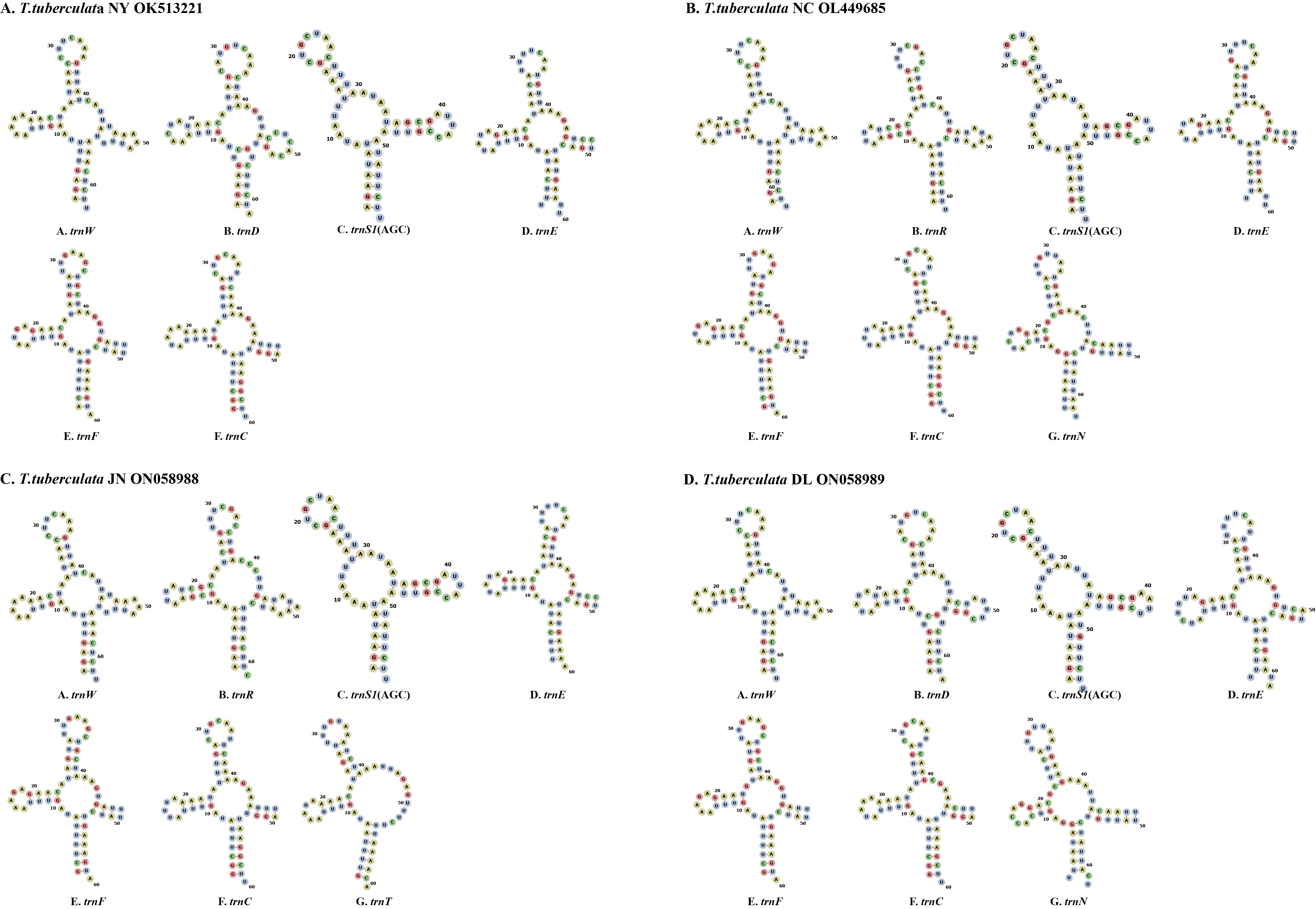

Supplement: Supplementary file 1 [file insects-13-00620-s001.zip › Figure S1. Trna.png]
